# Neurobiological Insights Into Cerebral Palsy: A Review of the Mechanisms and Therapeutic Strategies

**DOI:** 10.1002/brb3.70065

**Published:** 2024-10-08

**Authors:** Izere Salomon

**Affiliations:** ^1^ Department of General Medicine and Surgery University of Rwanda College of Medicine and Health Sciences Kigali Rwanda

**Keywords:** cerebral palsy | neurodevelopmental disorder | neuroplasticity | neuroprotection | neurorehabilitation | pediatric neurology

## Abstract

**Background:**

Cerebral palsy (CP) is a common neurodevelopmental disorder characterized by impaired mobility and posture caused by brain injury or abnormal development. CP relates to a variety of neurological mechanisms and pathways that impact the type and severity of motor disability, as well as comorbidities. The heterogeneity in clinical phenotype, pathogenesis, and etiology poses significant challenges for effective therapeutic intervention.

**Objectives:**

The review aims to provide a comprehensive analysis of the neurobiological mechanisms underlying CP and evaluate current and prospective therapeutic strategies, highlighting the necessity for targeted interventions to address the disorder's multifaceted nature.

**Methods:**

A thorough literature review was conducted, focusing on studies published in peer‐reviewed journals that explore the pathophysiological mechanisms, clinical interventions, and therapeutic strategies for CP.

**Results:**

The pathogenesis of CP involves a complex interplay of genetic, environmental, and perinatal factors leading to brain injury. Inflammatory processes, oxidative stress, and excitotoxicity are critical in CP development. Current therapeutic approaches primarily focus on symptom management through physical and occupational therapy, as well as pharmacological interventions. Emerging therapies, including anti‐inflammatory agents, antioxidants, and neuroprotective and neurotrophic agents, show potential but require further validation. Notably, although steroids provide anti‐inflammatory benefits, their use in pediatric patients raises concerns regarding long‐term adverse effects such as osteoporosis.

**Conclusion:**

Despite advances in understanding CP's neurobiological underpinnings, effective therapeutic targets remain elusive. A comprehensive approach addressing CP's heterogeneity is essential. Future research should emphasize in‐depth evaluations of the efficacy and safety of therapeutic agents, particularly in pediatric populations, to develop targeted and effective treatments for CP.

## Graphical abstract

Highlights the types, risk factors, and therapeutic strategies associated with cerebral palsy (CP). It emphasizes the complex interplay of genetic, environmental, and maternal factors, along with current therapeutic approaches aimed at managing symptoms and improving patient outcomes.

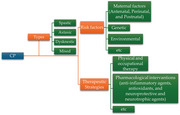

**Abbreviations**
CP: cerebral palsy
CT: computed tomography
DBS: deep brain stimulation
DNA: deoxyribonucleic acid
DTI: diffusion tensor imaging
EEG: electroencephalography
EMG: electromyography
EMGBF: electromyographic feedback
ERPS: event‐related protocol
fMRI: functional magnetic resonance Imaging
MEPs: motor‐evoked protocols
MRI: magnetic resonance imaging
MRS: magnetic resonance spectroscopy
NCS: nerve conduction studies
NCV: nerve conduction velocity
PET: Positron emission tomography
PVL: periventricular leukomalacia
SPECT: single‐photon emission computed tomography
tDCS: transcranial magnetic stimulation
TMS: transcranial magnetic stimulation

## 1 Introduction

The most frequent cause of developmental impairment, cerebral palsy (CP), affects 17 million people worldwide and almost 1 in 345 children in the United States (Cerebral Palsy Facts, and Statistics 2024; Chin, Johnson, and Hoon 2021; McIntyre et al. 2022). CP is a set of neurological illnesses caused by damage to or aberrant development of the brain during the prenatal or early postnatal period, leading to decreased motor function and posture (Vova 2022). It is a diverse disorder that can manifest in a range of ways, including different types and intensities of motor impairment and related comorbidities, such as behavioral, sensory, and cognitive issues (Cerebral Palsy 2024c).

**FIGURE 1:**
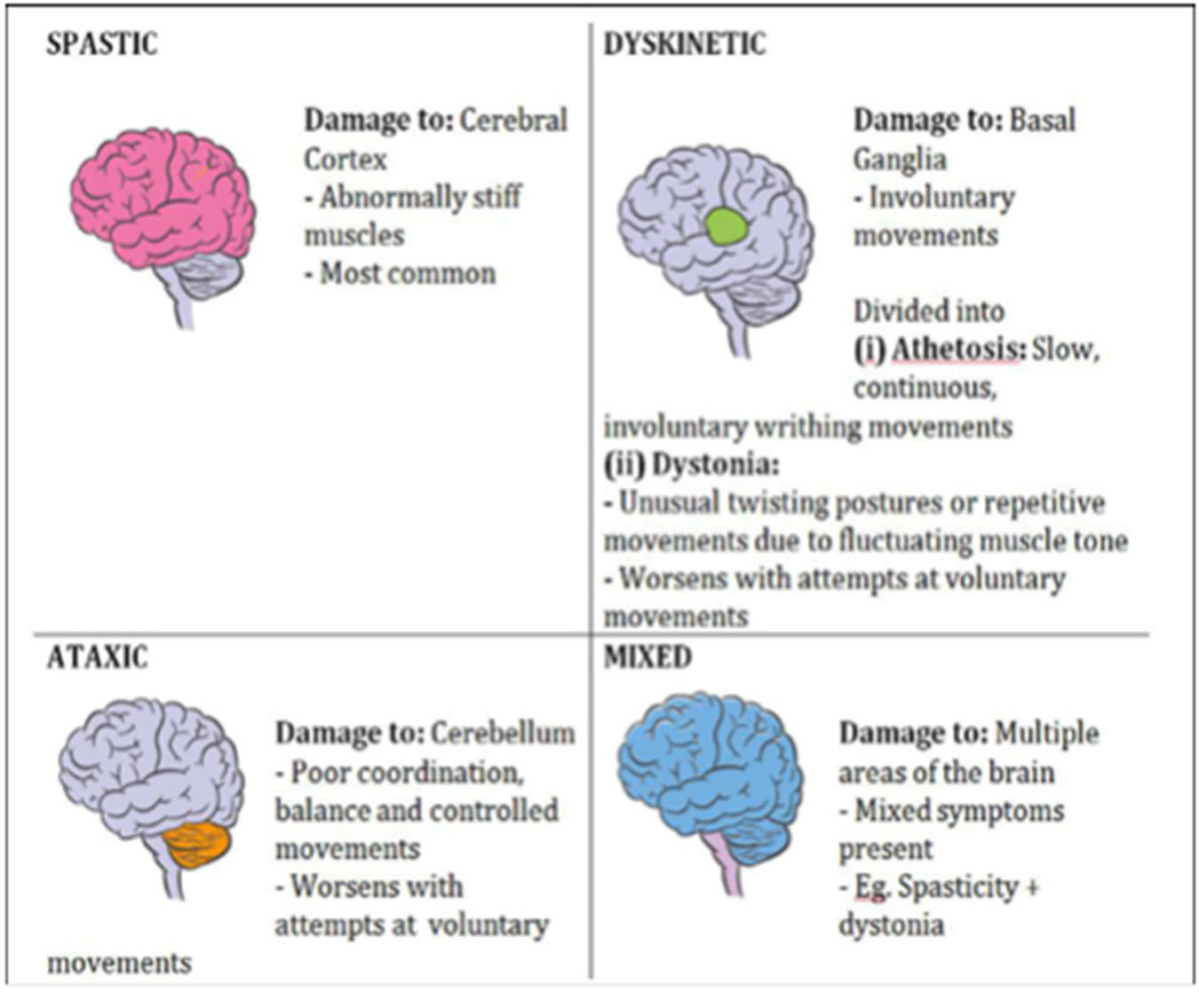
Depiction of various cerebral palsy (CP) forms on the basis of the type and location of brain injury (CDC 2023).

The complex and multifaceted neurobiological mechanisms behind CP entail interactions between genetic, environmental, and epigenetic factors that impact the shape and function of the growing brain (Fahey et al. 2017; Friedman et al. 2022; Lewis et al. 2021). The cerebral cortex, basal ganglia, and cerebellum—brain regions in charge of movement planning, execution, and coordination—are the regions most frequently damaged by CP (Cerebral Palsy 2024c). Damage to or disruption of these regions, as indicated in Figure 1, can result in different types of CP, including spastic, dyskinetic, and ataxic (CDC 2023).

The objectives of this review were to present the state of the art concerning the neurobiological causes of CP and to identify promising treatment targets that may improve the prognosis and quality of life of CP patients.

## 2 Etiology and Pathophysiology of Cerebral Palsy

**FIGURE 2:**
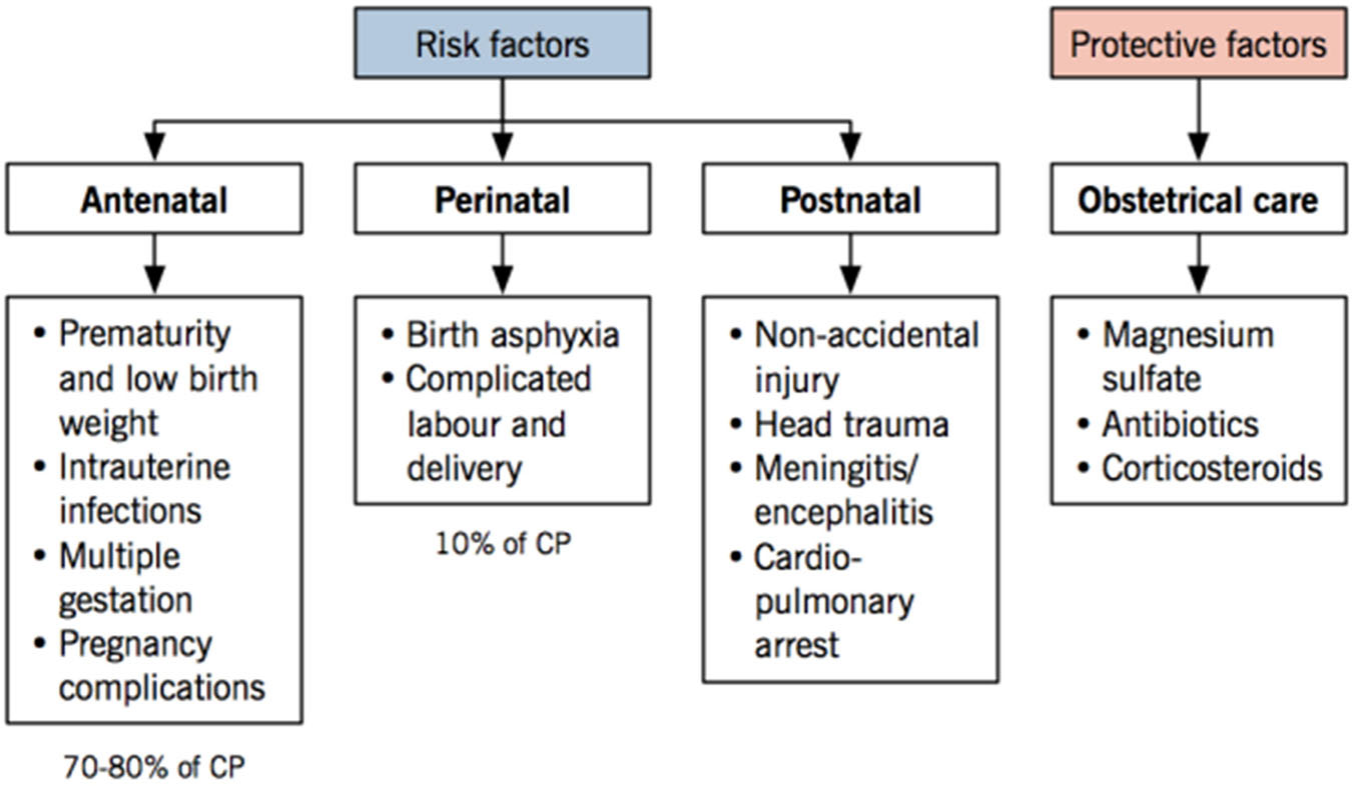
Risk factors linked to cerebral palsy development (Cerebral palsy 2012).

A variety of hereditary and environmental factors that impact the developing brain are involved in the multifactorial causation of CP. There are three groups of risk factors for CP: prenatal, perinatal, and postnatal (Figure 2) (Kurt 2016; Rosello et al. 2021). Prenatal factors, which occur before or during pregnancy, account for approximately 70%–80% of all CP cases (Wang, Bartell, and Wang 2017). Maternal diseases such as CMV, rubella, toxoplasmosis, and Zika virus can penetrate the placenta and result in congenital brain abnormalities or inflammation. Other prenatal variables include maternal diseases such as diabetes, hypertension, preeclampsia, and thyroid problems, all of which can have an impact on fetal growth and oxygen delivery (Kumar, Saadaoui, and Al Khodor 2022; Megli and Coyne 2022). Furthermore, genetic mutations, chromosomal abnormalities, and epigenetic alterations can predispose people to CP by altering the creation and function of neurons and glia (Lewis et al. 2021).

Factors related to pregnancy and childbirth constitute approximately 10%–20% of instances of CP, which includes a lack of brain oxygen, placental abruption, prolapses of the umbilical cord, or prolonged labor (Eunice Kennedy Shriver National Institute of Child Health and Human Development—NICHD 2024; Megli and Coyne 2022).

Birth trauma from instrumental delivery, breech presentation, or macrosomia can result in cerebral bleeding, a fractured skull, and nerve damage. These are examples of additional perinatal risks (Kumar, Saadaoui, and Al Khodor 2022). Approximately 10% of cases of CP are caused by postnatal causes, which occur after birth. Neonatal illnesses that can induce inflammation and damage to the brain include meningitis, encephalitis, and sepsis (NICHD—Eunice Kennedy Shriver National Institute of Child Health and Human Development 2024b). Hypoxic–ischemic encephalopathy, a brain injury caused by decreased blood flow and oxygen to the brain as a result of cardiac arrest, respiratory failure, or shock, is one of the other postnatal factors (NICHD—Eunice Kennedy Shriver National Institute of Child Health and Human Development 2024b). Prematurity and low birth weight are also important risk factors for CP because they impede brain development and increase the likelihood of brain injury.

A series of actions contribute to the pathophysiology of CP, disrupting normal brain development and function (Cerebral Palsy 2024c). Primary brain injury is the result of direct damage to brain tissues caused by mechanical, ischemic, or inflammatory factors. It can be caused by an initial insult that occurs during pregnancy, during childbirth, or after childbirth (NICHD—Eunice Kennedy Shriver National Institute of Child Health and Human Development 2024a, 2024b). Secondary brain injury involves gradual and progressive damage to brain tissue caused by oxidative stress, excitotoxicity, apoptosis, and inflammation. It can be induced by primary brain injury. Tertiary brain injury, described as the long‐term modification of brain structure and function as a result of compromised neurogenesis, synaptogenesis, myelination, and plasticity, can result from secondary brain injury (CDC 2023; Cerebral Palsy 2024b).

The cerebral cortex, basal ganglia, and cerebellum—all of which are important for motor control and coordination—are the brain areas most susceptible to CP (Cerebral Palsy 2024c). The outer layer of the brain that controls motor execution and planning is called the cerebral cortex. Increased muscular tone and stiffness are the hallmarks of spastic CP, which can be caused by damage to or abnormalities in the cerebral cortex (Cerebral Palsy 2024b; Rosello et al. 2021). A collection of subcortical nuclei called the basal ganglia is in charge of learning and motor control (Basal Ganglia 2024). Dyskinetic CP is a condition characterized by erratic muscle tone and involuntary movements that may be caused by damage to or dysfunction of the basal ganglia (Basal Ganglia Stroke 2024). The posterior region of the brain that controls balance and motor coordination is called the cerebellum. Ataxic syndrome may result from cerebellar damage or hypoplasia.

Potential treatment options that stop, lessen, or repair brain damage and dysfunction are provided by the pathophysiological pathways of CP (Cerebral Palsy 2024b). Anti‐inflammatory drugs, neuroprotective drugs, neurotrophic factors, stem cells, gene therapy, and other treatments are examples of these therapies. Nevertheless, research is currently ongoing to assess the safety and efficacy of these medicines, as well as the best time to provide them (El Ouaamari et al. 2023). Thus, more investigations are needed to clarify the molecular and cellular causes of CP and to create cutting‐edge, efficient treatments for CP (Patel 2005).

## 3 Neuroimaging and Neurophysiological Findings

Investigating the anatomical and functional abnormalities of the brain linked to CP can be facilitated by employing neuroimaging and neurophysiological approaches (Biasiucci, Franceschiello, and Murray 2019). Brain imaging can provide information about the type, location, and magnitude of brain abnormalities or lesions as well as the alterations in brain activity and connectivity caused by CP (Brammer 2009; Neuroimaging Techniques and What a Brain Image Can Tell Us 2024). Neurophysiology evaluates the health and functionality of the neural networks involved in motor control and execution in addition to measuring the electrical activity of the brain and muscles (Siddiqi et al. 2022).

Numerous modalities, including ultrasonography, CT, magnetic resonance imaging (MRI), PET, and SPECT, have been employed in neuroimaging investigations to assess brain structure and metabolism in people with CP (Burgos 2023; Kakkar et al. 2021). The most commonly used neuroimaging method is MRI, which may reveal details about the white and gray matter structures of the brain and has excellent spatial resolution and contrast. Additionally, functional imaging via MRI, such as magnetic resonance spectroscopy (MRS), diffusion tensor imaging (DTI), and functional magnetic resonance imaging (fMRI), can be performed on people with CP to reveal biochemistry, brain activation, and connectivity (fMRI [Functional MRI] 2024; Whitten 2012).

**FIGURE 3:**
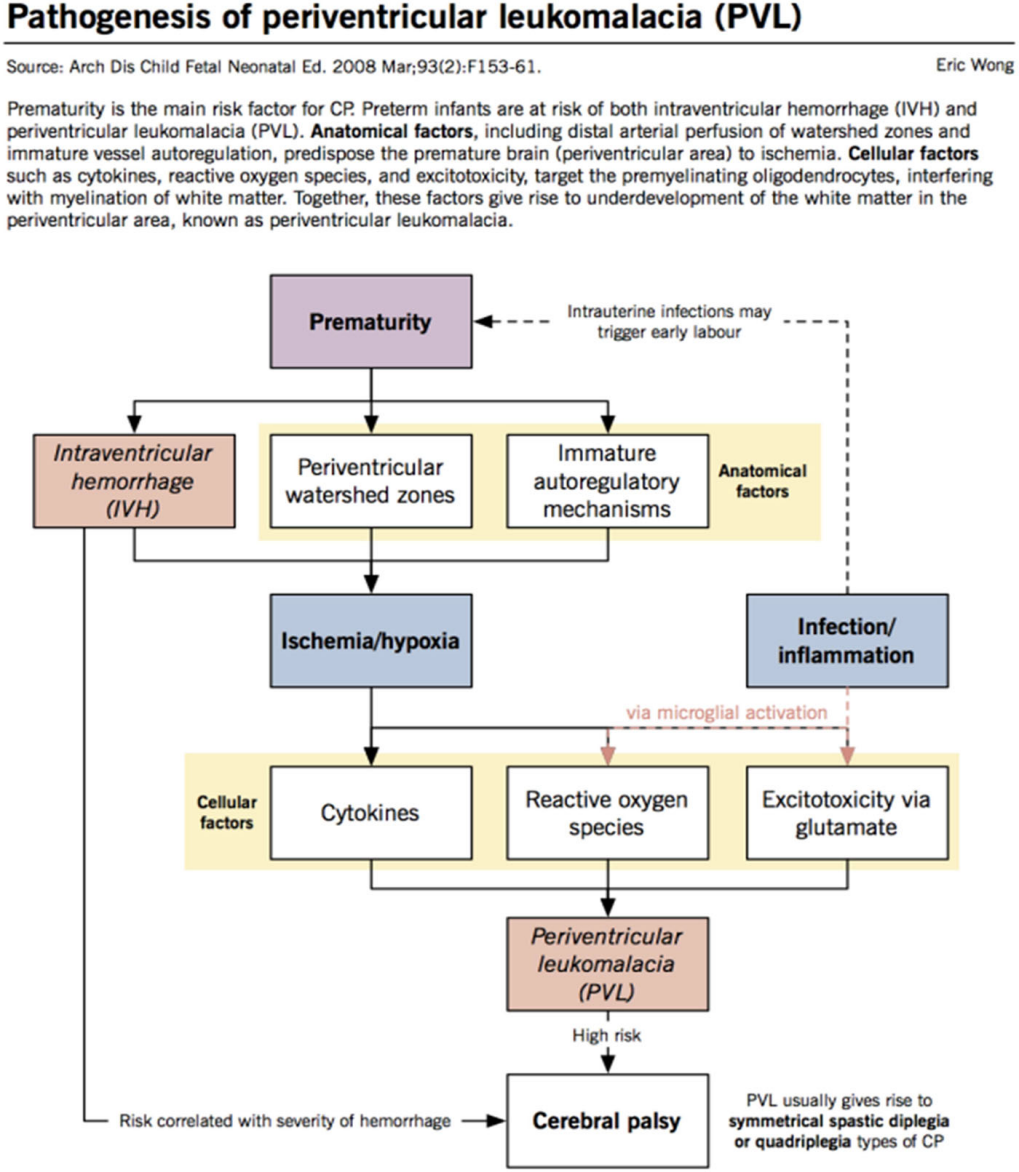
Pathogenesis of periventricular leukomalacia (PVL) (Khwaja and Volpe 2008).

According to neuroimaging research, the type, duration, and intensity of brain injury affect the patterns of abnormalities in the brains of CP patients (Hoon 2005). PVL, damage to the white matter around the ventricles, cerebral infarction, or the loss of blood supply to a portion of the brain are the most frequent MRI findings in CP (Figure 3) (Cerebral Palsy 2024c; Khan, Talat, and Malik 2022). These results are usually linked to spastic CP and demonstrate how susceptible the developing brain is to hypoxic–ischemic injury (Khan, Talat, and Malik 2022). Porencephaly, the development of cystic holes in the brain, schizencephaly, or abnormal clefting of the cerebral hemispheres are two additional radiological findings associated with CP (Kundu et al. 2020).

These results are typically connected to the hemiplegic CP and are indicative of disruptions in cortical organization and neuronal migration. Furthermore, neuroimaging studies have revealed a number of congenital brain malformations, including agenesis or hypoplasia of the brainstem, cerebellum, or corpus callosum, which can lead to different types of CP, including dyskinetic or ataxic CP (Kundu et al. 2020; Staudt 2013).

Studies using neuroimaging have also shown that CP affects both brain function and structure. According to functional imaging studies, people with CP have different patterns of brain activation and connectivity, which may be the result of maladaptive or compensatory changes in the brain networks responsible for motor control (Hirsch, Bauer, and Merabet 2015; Sasikumar and Strafella 2021). For example, during motor activities, fMRI studies have demonstrated that people with CP have decreased the activation of the primary motor cortex and increased activation of the cerebellum, premotor cortex, and supplementary motor area (Madkhali, Aldehmi, and Pollick 2022; Van Oostende et al. 1997). These results imply that to compensate for compromised primary motor cortex function, people with CP rely more on the cerebellum and secondary motor regions.

According to DTI studies, people with CP exhibit greater mean diffusivity and decreased fractional anisotropy in the corticospinal tract, which points to a loss of myelination and axonal integrity in the primary motor pathway (Bergamino, Walsh, and Stokes 2021; Hashem et al. 2020; Roberts, Mathias, and Rose 2016). These results are correlated with the severity of motor disability and spasticity in CP patients. According to MRS research, the brains of CP patients have different concentrations of metabolites, such as choline, lactate, and *N*‐acetyl aspartate, which indicate brain tissue energy metabolism, neuronal survival, and membrane turnover (Cebeci et al. 2022; Cerebral Palsy 2024c; Wang et al. 2023).

Electroencephalography (EEG), electromyography (EMG), transcranial magnetic stimulation (TMS), and nerve conduction studies (NCSs) are a few of the techniques used in neurophysiological studies to measure the electrical activity of the brain and muscles and evaluate the integrity and function of the neural pathways involved in motor control (Hill et al. 2016; Keser et al. 2022).

Epileptic seizures are common in people with CP, and an EEG approach can be used to determine their frequency and existence. EEG records the electrical potentials generated by the brain. Event‐related potentials (ERPs), or the brain's reactions to particular stimuli such as auditory, visual, or somatosensory inputs, can also be measured with EEG (Keser et al. 2022). ERPs can reveal details about a person with CP's cognitive abilities and sensory processing.

Electromyographic biofeedback (EMGBF) is a type of neurofeedback that gives people with CP real‐time feedback on their muscle activity and can help them improve their voluntary control and relaxation of their muscles. EMG is a technique that records the electrical activity of the muscles and can be used to measure the muscle tone, strength, and coordination of individuals with CP (Electromyography [EMG] 2018; Raez, Hussain, and Mohd‐Yasin 2006). TMS is a method that measures motor‐evoked potentials (MEPs), or muscle reactions to brain stimulation, by stimulating the brain with a magnetic coil (Kobayashi and Pascual‐Leone 2003).

The primary motor pathway that runs from the brain to the spinal cord is called the corticospinal pathway, and MEPs can tell us about its excitability, latency, and amplitude. The neural conduction velocity (NCV) is the speed at which an electrical signal travels along a peripheral nerve. It is measured via the NCS method, which stimulates peripheral neurons with electrical stimuli (Doyal, Schoenherr, and Flynn 2023). Peripheral nerves are branches of the spinal cord that connect to muscles and other bodily tissues. The NCV can provide information on the health and function of these nerves.

According to neurophysiological studies, people with CP exhibit aberrant patterns of muscle and brain activity, which are indicative of malfunctioning neural networks related to motor control. According to EEG research, people with CP have lower alpha activity and higher slow‐wave activity, which in turn suggests lower levels of alertness and attention in the brain (Brandenburg, Fogarty, and Sieck 2019; Trevarrow et al. 2021, 2022). EEG studies have shown that people with CP also exhibit aberrant ERPs, such as absent or delayed P300, which point to a brain that is less capable of processing sensory information and performing cognitive functions (Al‐Sulaiman 2001; Helfrich and Knight 2019).

According to EMG research, people with CP exhibit less reciprocal inhibition and greater muscular contraction, which suggests a poor coordination and regulation of muscle activity (Mohammadyari Gharehbolagh et al. 2023). Additionally, EMG studies have demonstrated that people with CP exhibit decreased or nonexistent EMGBF, which suggests a problem with voluntary control and muscular relaxation60 (Cappellini et al. 2020; Yamanaka, Horiuchi, and Nojima 2023). According to TMS investigations, people with CP have lower MEP amplitudes and elevated motor thresholds, which suggests that their corticospinal system is less efficient and less excitable (Jannati et al. 2023; Tekgul et al. 2020). Additionally, TMS investigations have demonstrated that people with CP have decreased intracortical facilitation and increased intracortical inhibition, indicating an imbalanced balance between the motor cortex's excitatory and inhibitory circuits (Badawy et al. 2012; Jannati et al. 2023). According to NCS research, people with CP have normal or slightly reduced NCV, indicating that their function is either moderately impaired or retained (Jonsson et al. 2021).

In addition to aiding in the diagnosis, prognosis, and treatment of CP, neuroimaging and neurophysiological studies in CP offer important insights into the anatomical and functional brain abnormalities associated with CP (Himmelmann et al. 2021). They can also be used to assess the safety and effectiveness of different interventions, including medication, surgery, and rehabilitative therapy, by tracking their effects on the brain and muscle activity of people with CP (Crosson et al. 2010). However, there are several obstacles that hinder neuroimaging and neurophysiological research in CP. These include the heterogeneity and variability of CP, the difficulty of obtaining accurate and valid measurements in patients with CP, and the moral and practical difficulties associated with invasive or costly procedures for patients with CP (Ekanem et al. 2020; Fleming et al. 2018; Hadders‐Algra 2014). Thus, additional research is needed to enhance the caliber and relevance of neuroimaging and neurophysiological investigations in CP and to combine the results from many modalities and disciplines to offer thorough and comprehensive knowledge of the condition.

## 4 Neurobiological Mechanisms of Motor Impairment

The primary neuronal channels that link the motor cortex to the spinal cord and regulate voluntary muscle action are called corticospinal pathways, and disruption of these pathways results in motor impairments in people with CP (Cerebral Palsy 2024c; Jaspers et al. 2016). The lateral corticospinal tract, which innervates the distal muscles of the limbs, and the anterior corticospinal tract, which innervates the proximal muscles of the trunk, constitute the corticospinal pathways (Corticonuclear and Corticospinal Tracts 2024; Javed, Reddy, and Lui 2024). Spasticity, dystonia, and other movement disorders are among the movement‐related deficits that can be caused by injury or malfunction to the corticospinal pathways (Javed, Reddy, and Lui 2024).

Approximately 80% of those with CP suffer from spasticity, the most prevalent motor impairment in CP (Cerebral Palsy 2024c). Spasticity is defined as an increase in muscle tone and resistance to passive stretch that is velocity dependent and results from the stretch reflex being hyperexcitable (Miller 2020). Spasticity can lead to discomfort, contractures, and deformities as well as reduce muscular strength, coordination, and range of motion. Additionally, it may disrupt the growth and operation of other systems, including the basal ganglia, cerebellum, and sensory systems (Rose, Papadelis, and Gaebler‐Spira 2020). Although the precise neurobiological mechanisms behind spasticity in CP remain unclear, both the peripheral and central components are thought to play a role. Changes in muscle characteristics, such as elevated rigidity, decreased flexibility, and modified fiber composition, are examples of peripheral factors (Albright 2023; Bar‐On et al. 2015). Changes in the brain and spinal cord, such as diminished inhibition, enhanced excitement, and modified plasticity, are essential components.

Approximately 15%–20% of people with CP have dystonia, a movement disorder that is characterized by involuntary, persistent, or intermittent muscle spasms that result in aberrant postures or motions. Dystonia is another prevalent motor disability in CP (van de Pol et al. 2022). Although it can affect any area of the body, the neck, trunk, and limbs are the most frequently affected, which may result in discomfort, exhaustion, and functional impairment (Dystonic Cerebral Palsy 2024; van de Pol et al. 2022). Additionally, spasticity and dystonia can coexist to produce a mixed form of CP. Although the exact neurobiological causes of dystonia in CP remain unclear, it is believed that the subcortical basal ganglia, which control the motor output of the cortex, are malfunctioning (Downs et al. 2019; Dystonic Cerebral Palsy 2024; van de Pol et al. 2022). Both direct brain damage and the indirect effects of brain injury, such as inflammation, oxidative stress, and hypoxia, can cause dysfunction of the basal ganglia (Yanagisawa 2018).

Ataxia, athetosis, chorea, ballismus, and tremor are additional movement‐related disabilities associated with CP (Cerebral Palsy 2024a; Coordination Disorders 2024; Sato 2020). The deficit of balance and coordination known as ataxia results from injury or malfunctioning of the cerebellum, the posterior region of the brain that controls movement timing and precision (Albright 2023; Sato 2020). The impairment of smooth and continuous motions known as athetosis is caused by injury to or malfunction of the globus pallidus, a portion of the basal ganglia that suppresses the brain's relay center, the thalamus. Chorea is the incapacity to move voluntarily owing to injury or malfunction of the putamen and caudate nucleus, two regions of the basal ganglia that support the thalamus (Sato 2020).

Ballismus is a disorder of proximal motion caused by injury to or malfunction of the subthalamic nucleus, a component of the basal ganglia that controls globus pallidus functioning (Rocha Cabrero and De Jesus 2024). The inability to move steadily is known as tremor, and it is caused by injury or malfunction to the brainstem, the area of the lower brain that connects to the spinal cord (Lenka and Jankovic 2021).

## 5 Emerging Therapeutic Targets

The difficult nature of CP therapy frequently necessitates a multidisciplinary strategy that includes medication, surgery, and rehabilitation techniques. These treatments, however, primarily target symptoms rather than the underlying neurological causes of CP. As a result, new and efficient treatment modalities that address the brain damage and dysfunction that cause CP are needed (Specific Therapeutic Interventions for Individuals with Cerebral Palsy 2024).

In CP patients, neuroprotective techniques are used to avoid or reduce primary and subsequent brain damage. Gene therapy, stem cell therapy, and hypothermia are some of these tactics. After a hypoxic–ischemic insult, hypothermia involves cooling of the body or head to decrease metabolic demand and the inflammatory response of the brain. One of the main causes of CP, hypoxic–ischemic encephalopathy, in neonates is improved neurologically, and neonatal mortality is decreased by hypothermia (Specific Therapeutic Interventions for Individuals with Cerebral Palsy 2024). To promote the repair and regeneration of injured brain tissue, stem cell treatment involves transplanting stem cells—such as neural, mesenchymal, and cord blood stem cells—into the brain or circulation. In certain clinical trials, stem cell treatment increases motor function, and neuroimaging results in children with CP (Physiotherapy Treatment Approaches for Individuals with Cerebral Palsy 2024).

To modify the expression of genes implicated in the pathophysiology of CP, therapeutic genes, such as neurotrophic factors, anti‐inflammatory medicines, and antiapoptotic agents, are delivered into the brain or circulation via gene therapy. Preclinical studies have shown that gene therapy improves both histopathological and motor function in animal models of CP (Fehlings et al. 2023).

Neuromodulation techniques are aimed at increasing or restoring the function of motor control–related neural pathways (Lewis et al. 2016). These methods include deep brain stimulation (DBS), TMS, and transcranial direct current stimulation (tDCS). tDCS modifies the excitability and plasticity of the cortex by applying a small electric current to the scalp (Lewis et al. 2016; Luan et al. 2014). In certain therapeutic trials, tDCS has been demonstrated to enhance cortical activation and motor performance in children with CP. TMS is the process of stimulating or inhibiting the cortex by applying a magnetic pulse to the scalp.

In certain therapeutic trials, TMS has been demonstrated to enhance corticospinal excitability and motor function in children with CP. To activate or inhibit certain brain regions, such as the cerebellum or basal ganglia, DBS entails implanting electrodes into the brain (Aviles‐Olmos et al. 2014; Koeglsperger et al. 2019). Some case reports have demonstrated that DBS enhances motor function and quality of life in children with CP.

Targeting the molecular and cellular pathways implicated in the pathogenesis of CP is the goal of emerging pharmaceutical treatments. Anti‐inflammatory, antioxidant, neuroprotective, and neurotrophic factors are some of these therapies (Landry and Gies 2008; Min and Lee 2022).

Anti‐inflammatory agents are medications that prevent the body from producing or acting upon inflammatory mediators, which include prostaglandins, nitric oxide, and cytokines and are implicated in brain damage and dysfunction in CP (French et al. 2017; Mallah et al. 2020). Steroids, nonsteroidal anti‐inflammatory medications, and minocycline96 are examples of anti‐inflammatory agents (Mallah et al. 2020). Antioxidants are medications that scavenge or neutralize reactive oxygen species, which in CP cause oxidative stress and damage to brain tissue. Examples of these species include superoxide, hydrogen peroxide, and hydroxyl radicals. Melatonin, vitamin C, and vitamin E are examples of antioxidants (Flieger et al. 2021; Halliwell 2024). Drugs known as neuroprotective medicines work to stop or lessen the neuronal loss or malfunction that occurs in CP (Mallah et al. 2020).

Calcium channel blockers, sodium channel blockers, and *N*‐methyl‐d‐aspartate receptor antagonists are examples of neuroprotective drugs. Proteins known as neurotrophic factors help neurons and glia survive, proliferate, and differentiate. Brain‐derived neurotrophic factors, nerve growth factors, and neurotrophic factors generated from glial cell lines are examples of neurotrophic factors (Neuroprotective Agent 2024).

These neurobiologically guided CP treatment strategies present encouraging paths toward enhancing the prognosis and quality of life of CP patients. To determine these strategies’ effectiveness, safety, and ideal parameters, more research is necessary, as they are still in the early phases of development (Faccioli et al. 2023). Furthermore, these methods will probably work better in conjunction with more traditional therapies, such as speech, occupational, and physical therapy, which work to improve the participation and functional skills of people with CP. To fully understand the synergistic effects and optimal use of these therapies for CP, more research is necessary.

## 6 Neurorehabilitation and Neuroplasticity

The goal of neurorehabilitation therapy is to improve a person with CP's functional abilities and engagement in a variety of domains, including movement, communication, cognition, and socialization (Novak 2016; Reid, Rose, and Boyd 2015). Physiotherapy, occupational therapy, speech therapy, and assistive technology are examples of neurorehabilitation interventions (Faccioli et al. 2023). By utilizing neuroplasticity—the brain's capacity to change and reorganize itself in response to experience and learning—neurorehabilitation therapies can help people with CP function better. By offering task‐specific, intense, and goal‐oriented training that challenges the person and engages their motivation and attention, neurorehabilitation therapies can promote neuroplasticity. By offering direction, reinforcement and feedback that improve learning and performance on an individual basis, neurorehabilitation therapies can also promote neuroplasticity (Puderbaugh and Emmady 2024; Reid, Rose, and Boyd 2015).

As neuroplasticity has the ability to repair or compensate for the brain damage and dysfunction that underlie CP, it is a promising therapeutic option for the management of CP (Puderbaugh and Emmady 2024; Reid, Rose, and Boyd 2015). The molecular and cellular pathways that mediate brain injury and healing can be modulated by a variety of strategies, including neuroprotective tactics, neuromodulation techniques, and newly developed pharmaceutical interventions (Novak 2016). A person's age, genetics, environment, and lifestyle can all have an impact on neuroplasticity, which in turn affects how their brain develops and functions in people with CP. It can be quantified by a range of methods that reveal anatomical and functional alterations in the brain and behavior of people with CP, including neuroimaging, neurophysiology, and behavioral examinations.

The principles of neuroplasticity and neurorehabilitation are complementary to one another and can help with CP management. Through neuroplasticity, neurorehabilitation can improve quality of life and results in people with CP. For the purpose of creating and assessing innovative and successful treatments for CP, neuroplasticity offers a scientific foundation as well as therapeutic targets (Neuroplasticity and Cerebral Palsy 2024). However, a number of obstacles still stand in the way of our ability to fully comprehend and apply neurorehabilitation and neuroplasticity in CP. These obstacles include the heterogeneity and variability of CP, the diversity and complexity of the neurobiological mechanisms underlying CP and the moral and financial ramifications of invasive or costly procedures for CP patients (Neuroplasticity and Cerebral Palsy 2024).

To provide a thorough and integrated treatment for CP, more research is therefore needed to improve our understanding of neurorehabilitation and neuroplasticity in CP and to integrate findings from many modalities and disciplines.

## 7 Innovations in Neuroregenerative Therapies

The goal of neuroregenerative treatments is to replace lost or injured brain tissue in the CP, hence restoring its structure and function (Te Velde et al. 2022). These treatments include extracellular vesicles, tissue engineering, nanotechnology, gene therapy, and stem cell therapy, among other regeneration techniques. For those with CP, neuroregenerative treatments may be able to lessen brain damage and encourage recovery (Physiotherapy Treatment Approaches for Individuals with Cerebral Palsy 2024). These treatments, however, are still in the experimental phase and face numerous difficulties, including questions about safety, efficacy, and ethics.

Undifferentiated stem cells with the ability to self‐renew and specialize into different cell types are transplanted into the brain or bloodstream of patients with CP as part of stem cell treatment (Te Velde et al. 2022). Numerous sources of stem cells, including neural stem cells, mesenchymal stem cells, induced pluripotent stem cells, embryonic stem cells, and umbilical cord blood cells, exist. Stem cell therapy stimulates angiogenesis, replaces lost cells, reduces inflammation, and provides trophic support to aid in the healing and regeneration of injured brain tissue. In certain clinical trials, stem cell treatment increases motor function, and neuroimaging results in children with CP (Te Velde et al. 2022).

The variability and heterogeneity of stem cell sources; the best method, dose, and time for delivering stem cells; the ability of transplanted cells to integrate and survive; and the possibility of tumorigenesis and immunogenicity of stem cells are some of the drawbacks of stem cell therapy (Aly 2020; Tang 2019).

Gene therapy involves injecting therapeutic genes—segments of DNA that code for a particular protein or function—into the bloodstream or brain of patients who have CP (Matharu and Ahituv 2020; Rittiner et al. 2022). Gene therapy has the ability to modify the expression of genes, including neurotrophic factors, anti‐inflammatory drugs, and antiapoptotic agents, that are implicated in the pathogenesis of CP (Rittiner et al. 2022). Gene therapy can also address genetic flaws or mutations, such as chromosomal abnormalities or epigenetic changes, that predispose people to CP. Numerous delivery systems, including viral, nonviral, and nanoparticle systems, can be used for gene therapy. Preclinical studies have shown that gene therapy improves both histopathological and motor function in animal models of CP.

The effectiveness and selectivity of gene delivery, the length and control of gene expression, and the moral and safety concerns around gene manipulation are some of the difficulties that gene therapy also faces (Gonçalves and Paiva 2017; Rittiner et al. 2022).

The goal of other regeneration techniques is to develop artificial or biological structures that can support or replace damaged brain tissue in CP patients. Tissue engineering, nanotechnology, and extracellular vesicles are some of these tactics (Liu, Xia, and Czernuszka 2007). The process of creating three‐dimensional scaffolds that resemble the extracellular matrix and offer an ideal environment for cell growth and differentiation is known as tissue engineering (Howard et al. 2008).

Bioengineered tissues or organs that can be inserted into the brain can be produced by tissue engineering in conjunction with stem cells or growth factors. The manipulation of matter at the nanoscale level to produce new materials or gadgets that communicate with biological systems is known as nanotechnology. Nanotechnology has the potential to monitor or stimulate brain function, as well as transfer medications, genes, or cells to the brain (Han et al. 2020; Howard et al. 2008). Membrane‐bound particles, known as extracellular vesicles, are secreted by cells and contain a variety of biomolecules, including lipids, proteins, and nucleic acids. Extracellular vesicles regulate cellular processes and act as mediators of intercellular communication. Therapeutic chemicals can be delivered to the brain via extracellular vesicles, which can be produced from stem cells or other sources (Gomzikova, James, and Rizvanov 2022; Guo et al. 2023).

Neuroregenerative therapies are innovative and exciting approaches that can transform CP management. However, these therapies are still in the experimental phase, and more evidence and research are needed to establish their feasibility, efficacy, and safety. Moreover, these therapies are likely to be more effective when combined with conventional interventions, such as physiotherapy, occupational therapy, and speech therapy, which aim to increase the functional abilities and participation of individuals with CP. Therefore, further research is needed to explore the synergistic effects and best practices of these therapies for CP (Physiotherapy Treatment Approaches for Individuals with Cerebral Palsy 2024; Specific Therapeutic Interventions for Individuals with Cerebral Palsy 2024).

## 8 Challenges and Future Directions

Because of its complexity and variety, CP poses a great barrier in identifying the precise neurobiological mechanisms underlying each individual case. It is difficult to customize neurobiological therapies for specific cases because of this intricacy. Furthermore, before being widely used in clinical settings, the field of neurobiological therapies for CP is still in its infancy and faces a number of ethical, legal, and technical challenges. Owing to the possibility of unforeseen consequences for the brain and other organs, monitoring the long‐term safety and effectiveness of these therapies is imperative.

In the future, more investigations are needed to clarify the neurological processes that underlie the many forms and subtypes of CP. The development of treatment plans requires the identification of biomarkers and indicators of brain injury and recovery. Furthermore, neurobiological therapies for CP must be developed and optimized, with the best possible timing, dose, combination, and delivery strategies identified. To assess the cost and effectiveness of these therapies in relation to current standards of care, extensive and prolonged clinical trials are necessary. Additionally, a promising direction for future studies is investigating how neuroplasticity and neurorehabilitation can improve functional ability and engagement in people with CP.

Creating novel approaches to activate and utilize the brain's capacity for learning and adaptability may enhance the results for people with CP. By addressing the complex and dynamic characteristics of CP, these future directions hope to pave the way for more thorough and efficient treatment plans in the area of CP‐related neurobiological insights. Improving the knowledge and treatment of CP through neurobiological discoveries can result in better results and a greater quality of life for those who are impacted by this condition by tackling these obstacles and moving in these new directions.

## Author Contributions

**Izere Salomon**: conceptualization, investigation, project administration, writing–original draft, writing–review and editing, validation, methodology.

## Conflicts of Interest

The author declares no conflicts of interest.

### Peer Review

The peer review history for this article is available at https://publons.com/publon/10.1002/brb3.70065.
